# The Guanylate Cyclase C—cGMP Signaling Axis Opposes Intestinal Epithelial Injury and Neoplasia

**DOI:** 10.3389/fonc.2018.00299

**Published:** 2018-08-06

**Authors:** Jeffrey A. Rappaport, Scott A. Waldman

**Affiliations:** Department of Pharmacology and Experimental Therapeutics, Thomas Jefferson University, Philadelphia, PA, United States

**Keywords:** guanylate cyclase C, cGMP, intestinal epithelium, colorectal cancer, microbiome, DNA repair, inflammation, cancer prevention

## Abstract

Guanylate cyclase C (GUCY2C) is a transmembrane receptor expressed on the luminal aspect of the intestinal epithelium. Its ligands include bacterial heat-stable enterotoxins responsible for traveler's diarrhea, the endogenous peptide hormones uroguanylin and guanylin, and the synthetic agents, linaclotide, plecanatide, and dolcanatide. Ligand-activated GUCY2C catalyzes the synthesis of intracellular cyclic GMP (cGMP), initiating signaling cascades underlying homeostasis of the intestinal epithelium. Mouse models of GUCY2C ablation, and recently, human populations harboring GUCY2C mutations, have revealed the diverse contributions of this signaling axis to epithelial health, including regulating fluid secretion, microbiome composition, intestinal barrier integrity, epithelial renewal, cell cycle progression, responses to DNA damage, epithelial-mesenchymal cross-talk, cell migration, and cellular metabolic status. Because of these wide-ranging roles, dysregulation of the GUCY2C-cGMP signaling axis has been implicated in the pathogenesis of bowel transit disorders, inflammatory bowel disease, and colorectal cancer. This review explores the current understanding of cGMP signaling in the intestinal epithelium and mechanisms by which it opposes intestinal injury. Particular focus will be applied to its emerging role in tumor suppression. In colorectal tumors, endogenous GUCY2C ligand expression is lost by a yet undefined mechanism conserved in mice and humans. Further, reconstitution of GUCY2C signaling through genetic or oral ligand replacement opposes tumorigenesis in mice. Taken together, these findings suggest an intriguing hypothesis that colorectal cancer arises in a microenvironment of functional GUCY2C inactivation, which can be repaired by oral ligand replacement. Hence, the GUCY2C signaling axis represents a novel therapeutic target for preventing colorectal cancer.

## Introduction

Constituting the largest interface with non-sterile material from the outside world, the intestinal epithelium regulates fluid and nutrient transport, hosts commensal flora, and protects against infiltration by toxins and pathogenic organisms that pass through the digestive tract (1). These functions are accomplished by a single-cell layer of columnar epithelial cells, which form a mechanical barrier dividing the systemic compartment from the turbulent gut lumen. Insults from the lumen induce continuous epithelial cell turnover, requiring tremendous regenerative capacity, with as many as 10^11^ gut epithelial cells replaced each day (2). This proliferative status predisposes the epithelium to neoplastic transformation, arising from corruption of circuits that normally maintain epithelial homeostasis.

**Figure 1:**
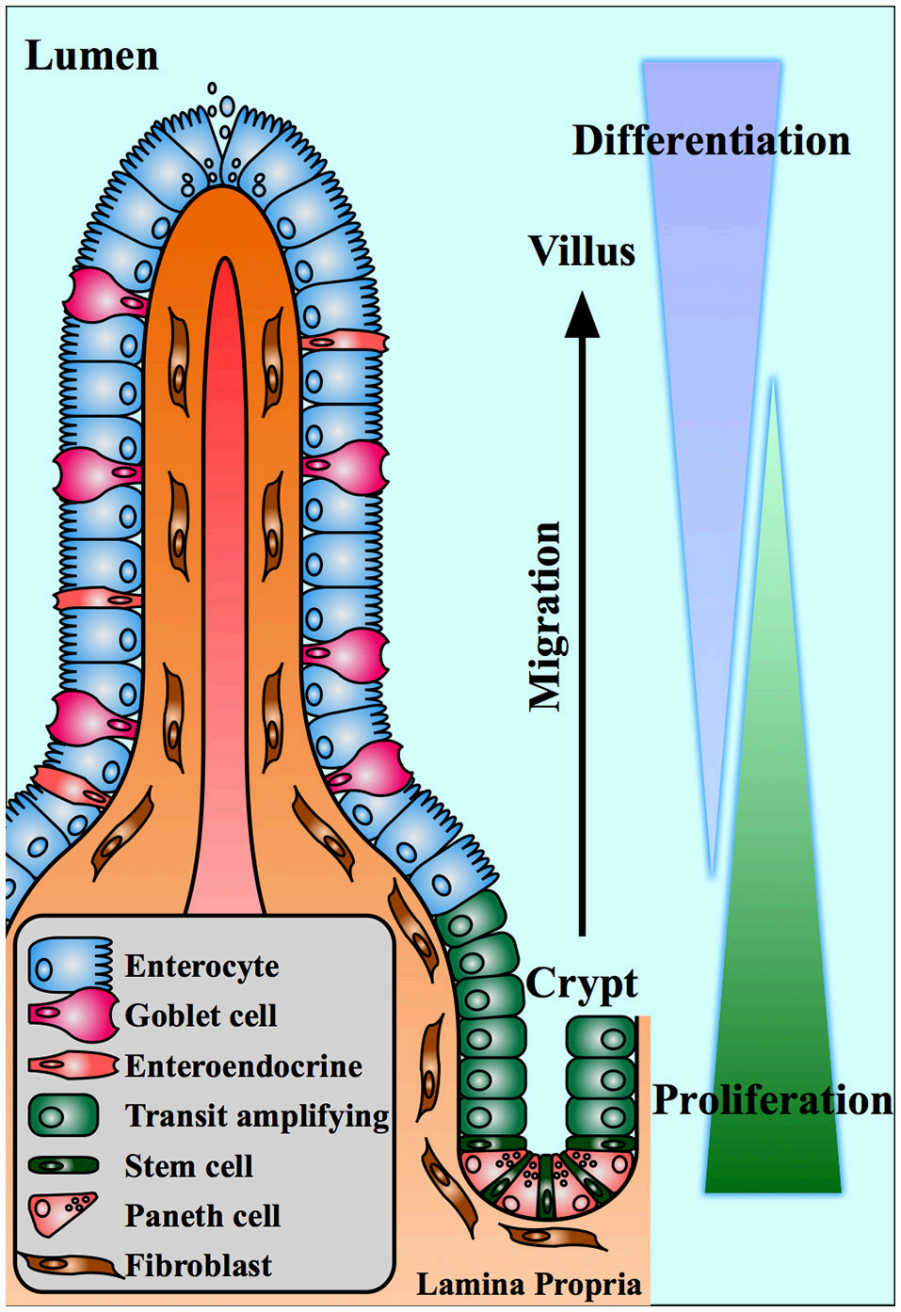
The intestinal crypt-villus axis. The small intestinal epithelium is organized into villus projections into the gut lumen and crypt invaginations into the lamina propria. Stem cells at the base of the crypt produce proliferative daughter cells that give rise to differentiated cells of the villus. Epithelial cells migrate from the proliferating compartment toward the tip of the villus where they undergo apoptosis.

The healthy epithelium exhibits a highly organized structure (Figure 1). Epithelial cells are polarized such that their apical surface faces the intestinal lumen, engaging in nutrient absorption and fluid secretion. The basolateral surface rests on a basement membrane and interfaces with the supportive stroma, vasculature, and mesenchymal cells of the underlying lamina propria. A network of junctional complexes stiches adjacent epithelial cells together, restricting paracellular transport between the mucosal surface, and subepithelial tissue (3). To increase absorptive surface area, the small intestinal epithelium is organized vertically with invaginations into the mucosa, called crypts, and projections into the lumen, called villi. In contrast, the surface of the large intestine is relatively smooth (lacking villi), with deep mucus-secreting crypts, enabling fluid absorption and stool transit.

This crypt-villus axis is a physiologically unique structure, characterized by continuous cell proliferation and turnover. At the base of the crypt, long-lived stem cells give rise to rapidly proliferating daughter cells, which differentiate into specialized epithelial cell subtypes (4). These cells migrate upwards from crypt to villus, differentiating into nutrient-absorbing enterocytes (the majority of the epithelial population), mucus-secreting goblet cells, and hormone secreting enteroendocrine cells (2). Another cell type, Paneth cells, migrate downward into the crypt, where they nourish the stem cells and secrete antimicrobial compounds into the lumen (5, 6). Terminally differentiated villus cells persist only 3–5 days, over which time they migrate to the tip of the villus, undergo apoptosis, and slough off into the fecal stream (2).

Tight homeostatic control of the circuits regulating cell division, differentiation, migration, and apoptosis, are critical to maintain the barrier integrity, secretory, and absorptive activity of the intestinal mucosa. The intestinal epithelial receptor, guanylate cyclase C (GUCY2C), and its cyclic nucleotide second messenger, cyclic guanosine monophosphate (cGMP), play a critical role in the maintenance of mucosal homeostasis, with GUCY2C being considered an emerging guardian of intestinal integrity. Identified nearly 30 years ago as a signaling network hijacked by diarrheagenic bacteria to stimulate intestinal secretion (7), cGMP signaling in the intestine is now recognized to underlie many homeostatic functions required for epithelial health. As such, dysregulation of cGMP signaling contributes to intestinal diseases including bowel transit disorders, inflammatory bowel disease, and cancer (8–11). We will briefly discuss the key players responsible for the generation, effector function, and degradation of cGMP in the intestine, followed by their contribution to intestinal physiology and disease. Finally, we will conclude with current approaches to targeting this axis for cancer prevention.

## Components of the intestinal cGMP signaling axis

**Figure 2:**
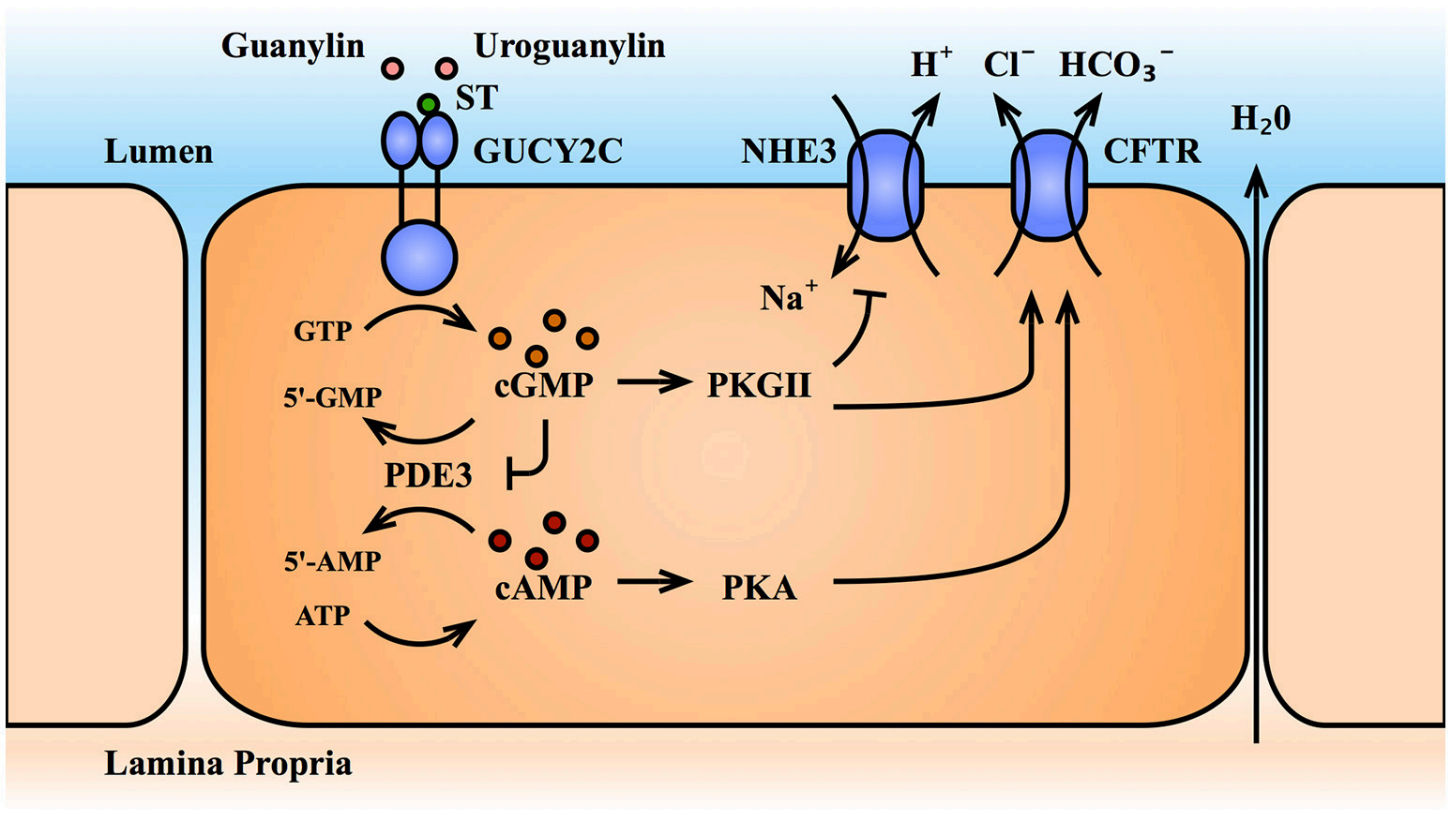
The GUCY2C-cGMP signaling axis and intestinal fluid secretion. Activation of the receptor GUCY2C by its ligands increases intracellular cGMP levels. The cGMP effector, PKGII, inhibits sodium ion absorption by the NHE3 transporter, and promotes anion secretion by the CFTR transporter, producing an electrolyte and fluid gradient into the gut lumen. Through inhibition of the dual-specificity phosphodiesterases, PDE3, cGMP accumulation also cross-activates cAMP/PKA signaling, which further potentiates CFTR. ATP, adenosine triphosphate; cAMP, cyclic adenosine monophosphate; CFTR, cystic fibrosis transmembrane conductance regulator; cGMP, cyclic guanosine monophosphate; GTP, guanosine triphosphate; GUCY2C, guanylate cyclase C; NHE3, Na^+^/H^+^ exchanger III; PDE3, phosphodiesterases III; PKA, protein kinase A; PKGII, protein kinase G II; ST, bacterial heat-stable enterotoxin; 5′-AMP, 5′-adenosine monophosphate; 5′-GMP, 5′-guanosine monophosphate.

Guanylate cyclases are a ubiquitous class of enzymes that catalyze the cyclization of the purine nucleotide guanosine triphosphate (GTP) to the second messenger, cGMP (12, 13). They are broadly classified by intracellular localization, residing in either the particulate (membrane-bound) or soluble (cytosolic) fractions of the cell. Depending on the isoform, guanylate cyclases are activated by an array of signals including peptide ligands, Ca^2+^ transients, and nitric oxide. In turn, cGMP effectors include cGMP-dependent protein kinases (PKGs), cGMP-gated ion channels, and phosphodiesterases (PDEs). The spatiotemporal parameters of intracellular cGMP transients are a function of synthesis by guanylate cyclases and degradation by phosphodiesterases. In the spirit of brevity, we refer readers to thorough reviews of guanylate cyclase signaling (12, 14). Here, we will focus on the key elements of cGMP signaling in the intestinal epithelium and their canonical role in fluid secretion (Figure 2).

### Guanylate cyclase C (GUCY2C)

The GUCY2C isoform belongs to the particulate family of guanylate cyclases. While other cyclases (including soluble guanylate cyclase and the particulate guanylate cyclases A and B) have a widespread tissue distribution, GUCY2C is largely restricted to the intestinal tract (15, 16). It is expressed as a homodimer on the apical brush border of intestinal epithelial cells from the duodenum to the rectum, with its ligand-binding extracellular domain facing the intestinal lumen and its intracellular catalytic domain facing the cytosol (12, 14). GUCY2C was initially characterized as the receptor for the bacterial heat-stable enterotoxin, ST, the causative agent of traveler's diarrhea (7, 17). Extracellular ST binding activates the catalytic domain, generating intracellular cGMP. In turn, cGMP signaling canonically drives phosphorylation and translocation of the cystic fibrosis transmembrane conductance regulator (CFTR) to the cell surface, triggering Cl^−^ and HCO${}_{3}^{-}$ efflux into the intestinal lumen (18–20). Additionally, cGMP signaling inhibits the apical Na^+^/H^+^ exchanger 3 (NHE3), preventing Na^+^ absorption from the lumen (18–20). The combined electrolyte efflux and retention in the lumen produces an osmotic gradient that drives fluid secretion and, in the pathological scenario, secretory diarrhea. Given this secretory function, GUCY2C has emerged as an attractive target for the treatment of constipation syndromes (21, 22). Two GUCY2C agonists recently received FDA-approval for the treatment of chronic idiopathic constipation and constipation-predominant irritable bowel syndrome: linaclotide (*Linzess*™) (23–25) an ST analog, and plecanatide *(Trulance*™*)* (26, 27) an analog of the endogenous GUYC2C ligand, uroguanylin (discussed below). Efficacy and tolerability of these agents was recently summarized (28).

### GUCY2C ligands

**Figure 3:**
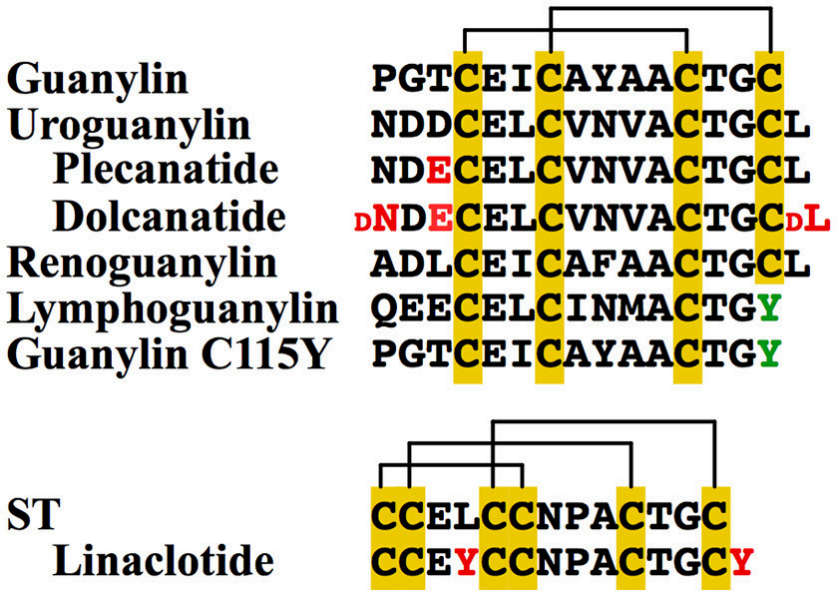
GUCY2C Ligands. The four members of the guanylin protein family are guanylin and uroguanylin, found in humans, and renoguanylin and lymphoguanylin, found in other species. While the first three have a structural conformation characterized by two disulfide bonds (yellow), lymphoguanylin lacks the C-terminal cysteine (green), and therefore has only one disulfide bond. A mutant guanylin (C115Y) similarly has only one disulfide bond and lower potency of GUCY2C activation. In contrast, bacterial ST is characterized by a rigid structure with three disulfide bonds. Synthetic GUCY2C agonists include plecanatide and dolcanatide (uroguanylin analogs), and linaclotide (ST analog). Amino acid differences between these analogs and the parent compound are shown in red.

Ligands of GUCY2C include the aforementioned ST, of bacterial origin, and the two endogenous peptides, uroguanylin and guanylin, secreted by the epithelium of the human small and large bowel, respectively (Figure 3) (29, 30). Two additional guanylin species of non-human origin, lymphoguanylin and renoguanylin, have been isolated from the American opossum (*D. virginiana*) and the European eel (*A. japonica*), respectively (31–33). The human intestinal guanylins are synthesized as propeptides by epithelial cells of secretory lineages (34–37), and processed to their mature, biologically-active, 16-mer (uroguanylin), or 15-mer (guanylin) forms. The propeptide sequence is thought to shield the site of ligand receptor interaction, although a precise role for the pro-sequence and the steps in peptide maturation remains unresolved (38). Structurally, these peptides are characterized by disulfide bridges (three for ST and two for the guanylins), which confer stability and resistance to denaturation (hence the name “heat-stable” enterotoxin) (14, 39, 40). Although the peptides share a high degree of sequence similarity, uroguanylin is more potent at acidic pH. In uroguanylin, two N-terminal aspartic acid residues were shown to act as an acidic switch, altering the protein conformation and enhancing ligand-receptor affinity 100-fold at pH 5 vs. pH 8 (40, 41). However, recent molecular dynamics simulations of lymphoguanylin suggest that the hydrophobic core, rather than the acidic N-terminal residues, controls peptide conformation (42). Despite our evolving understanding of the molecular behavior of these peptides, it is clear that differences in pH sensitivity of the ligands parallel their expression profiles along the intestinal axis, with uroguanylin expressed in the acidic environment of the duodenum, and guanylin expressed in the neutral environment of the colorectum (22).

Guanylin peptides have gained increasing attention as drug templates for the treatment of gastrointestinal disorders. In addition to the aforementioned linaclotide and plecanatide, both already FDA-approved, a second uroguanylin analog, dolcanatide has shown promise in ameliorating intestinal inflammation in rodent models (43, 44). From a production perspective, guanylin peptide analogs can be challenging to synthesize due to the presence of multiple cysteine bonds. Interestingly, lymphoguanylin and a recently-identified mutant form of human guanylin (C115Y) harbor a C-terminal tyrosine residue in place of the typical cysteine, eliminating one disulfide bridge in these species and lowering their potency to activate GUCY2C (Figure 3) (45). Recent single nucleotide polymorphism and structural analyses of guanylin peptide variants have lent insights into features that can be exploited to develop new compounds in this growing class of pharmaceutics (42, 45, 46).

### cGMP-dependent protein kinases

The primary effectors of cGMP are the cGMP-dependent protein kinases (PKGs), PKGI, and PKGII. PKGs belong to the serine/threonine class of protein kinases and consist of three domains: (1) an N-terminal domain necessary for homodimerization, autoinhibition, and subcellular localization, (2) a regulatory domain consisting of two cGMP binding pockets, and (3) a catalytic domain containing the ATP and substrate-binding pockets (47). cGMP binding drives a conformation change that releases the catalytic domain from the inhibitory N-terminal domain, enabling kinase activity. Both PKG isoforms are expressed in tissues throughout the body, including the intestine. PKGI is present in smooth muscle cells, where it regulates intestinal contractility (48), while PKGII is the predominant cGMP effector in the intestinal epithelium, where it regulates luminal fluid secretion (47, 49, 50). Tethered to the apical plasma membrane, cGMP-activated PKGII canonically phosphorylates CFTR and NHE3 to promote fluid and electrolyte efflux (51, 52).

### Phosphodiesterases

The cyclic nucleotides cGMP and cAMP are degraded to 5′-GMP and 5′-AMP by a family of enzymes called phosphodiesterases (PDEs). Eleven PDEs have been identified, each with varying tissue distribution, subcellular localization, and affinity for cGMP and cAMP. For example, PDE-4,−7, and−8 have higher affinity for cAMP, PDE-5,−6, and−9 for cGMP, and PDE-1,−2,−3,−10, and−11 hydrolyze both (53–55). PDEs that are expressed by the intestinal epithelium and contribute to cGMP hydrolysis include PDE-1,−2,−3,−5,−9 (53, 56, 57), and recently PDE10 (58). The extent to which epithelial-expressed PDEs with higher cAMP affinity [such as PDE4 (57)] modulate cGMP signaling remains unclear. However, cAMP and cGMP effectors converge on several physiological endpoints, including CFTR phosphorylation and fluid secretion. cGMP elevation indirectly potentiates cAMP effectors by occupying dual-specificity PDEs, thereby slowing degradation of cAMP. Additionally, some PDEs contain regulatory binding sites for cGMP that potentiate (PDE2 and 5) or inhibit (PDE3) cyclic nucleotide degradation. Hence, PDEs contribute a level of complexity to cyclic nucleotide signaling, particularly through cAMP-cGMP cross talk. These interactions remain to be comprehensively evaluated in the intestine, and are an area of significant interest.

## cGMP signaling and intestinal homeostasis

The most apparent role of cGMP signaling in the intestine can be appreciated from human populations harboring mutations in GUCY2C, resulting in hyper- or hypo-secretion syndromes. In the recently described familial GUCY2C diarrhea syndrome (FGDS), a single missense mutation in the catalytic domain of GUCY2C produces hyperactivation of the receptor in response to ligand (9, 59, 60). This rare autosomal dominant disorder (initially reported in 32 members of a Norwegian family) is clinically characterized by loose stools, inflammation resembling irritable bowel disease with diarrhea (IBS-D), a doubling of intestinal transit time, and elevated intestinal pH. GUCY2C-deactivating mutations have also been reported, including missense mutations in the ligand-binding and catalytic domains, and nonsense mutations eliminating the catalytic domain entirely (10, 61). These autosomal recessive disorders, reported in Bedoin and Lebanese families, produce meconium ileus (neonatal intestinal obstruction) due to GUCY2C insensitivity to its ligands, diminished epithelial cGMP, and diminished CFTR-mediated intestinal secretion.

These findings in humans mimic secretory defects observed in mice lacking components of the cGMP signaling axis. For example, GUCY2C^−/−^ (62, 63) and PKGII^−/−^ mice (50) are insensitive to ST-mediated intestinal fluid secretion. Guanylin deficient mice also have altered colonic electrolyte transport (64). Given the small population of humans harboring GUCY2C mutations, knockout mice have proven invaluable to the identification of the more subtle functions of cGMP in intestinal homeostasis, to be described below.

### cGMP, epithelial proliferation, and differentiation along the crypt-villus axis

Intestinal epithelial renewal requires a continuous supply of new cells produced by proliferation in the crypt. This renewal is regulated by a signaling cascade controlled by the extracellular ligand, Wnt, and its downstream transcriptional effector, β-catenin (65). In the differentiated villus, extracellular Wnt expression is low. In this context, cytosolic β-catenin enters a multi-protein complex stabilized by the scaffold proteins, adenomatous polyposis coli (APC) and axin. There, serine/threonine kinases, casein kinase 1a and glycogen synthase kinase 3, phosphorylate β-catenin, marking it for poly-ubiquitination by the β-TrCP E3 ubiquitin ligase and degradation by the proteasome. The presence of extracellular Wnt blocks this process. Wnt binds to its cell surface receptor, Frizzled, and co-receptor, LRP, which recruit axin to the plasma membrane to destabilize the destruction complex. This allows β-catenin to accumulate, translocate to the nucleus, associate with the T-cell factor (TCF) family of nuclear transcription factors, and activate a transcriptional program driving proliferation (66–69). Wnt hormones are secreted at the base of the intestinal crypt, providing a local niche conducive to stem cell renewal and epithelial proliferation (5, 65). The intestinal stem cell, identified only 10 years ago as the LGR5^+^ crypt base columnar cell (4), gives rise to daughter cells that populate the transit amplifying zone of the crypt. As these cells proliferate, migrate up the crypt, and leave the stem cell niche, Wnt tone diminishes and is replaced by Hedgehog and bone morphogenic protein (BMP) cascades, which support senescence and differentiation into the various specialized cells of the mature villus (70–72).

The balance of signaling promoting and opposing intestinal Wnt signaling is essential for life. Elimination of Wnt signaling in mice through disruption of β-catenin, TCF, its downstream target c-myc, or overexpression of the Wnt inhibitor dickkopf, results in crypt loss and fatal intestinal damage (73–76). Conversely, uncontrolled Wnt signaling underlies the majority of colorectal cancers. Spontaneous mutations inactivating the tumor suppressor, APC, or stabilizing oncogenic β-catenin represent the most common (>80%) driving mutations of sporadic colon cancer (77). APC is a prototypical tumor suppressor, where an initial spontaneous mutation produces allelic heterozygosity and cancer susceptibility. Loss of the remaining allele (loss of heterozygosity) eliminates APC from the β-catenin destruction complex, enabling uncontrolled β-catenin-driven transcription and tumorigenesis. This paradigm is most dramatic in patients with the hereditary cancer syndrome familial adenomatous polyposis (FAP), who harbor a germline mutation in one allele of APC and develop hundreds of adenomas throughout the colorectum by age 40 (77). This effect is mimicked in the widely-studied APC^min/+^ mouse, the first mouse model of intestinal cancer, which harbors a truncating germline mutation in one allele of APC and develops multiple intestinal polyps (78, 79).

cGMP signaling opposes intestinal proliferation and promotes differentiation. Genetic elimination of GUCY2C, its ligand guanylin, or the cGMP effector PKGII results in intestinal crypt hyperplasia, characterized by increased crypt length and expansion of the proliferating compartment of transit-amplifying cells (measured by the number of PCNA and Ki67-positive cells) (80–82). In turn, differentiated cells of the secretory lineage, including goblet, Paneth, and enteroendocrine cells, are lost (81, 82). Interestingly elimination of GUCY2C also changes the stem cell compartment, producing endoplasmic reticulum stress in the crypt and shifting the balance of stem cells from canonical LGR5^+^ cells, to reserve BMI1^+^ cells, which normally remain in a quiescent state and repopulate the crypt upon injury (83). Corresponding with these changes, silencing GUCY2C increases tumorigenesis in APC^min/+^ mice and mice exposed to the mutagens, azoxymethane or N-nitroso-N-methylamine, reflecting loss of epithelial cGMP (84, 85). These findings suggest that cGMP signaling opposes the events required for transformation by restricting proliferation and promoting differentiation along the crypt-villus axis. Interestingly, the absence of cGMP signaling is insufficient to induce tumorigenesis, but may instead create a selective advantage for transformed cells to proliferate and develop tumors.

Several studies demonstrate that cGMP opposes proliferation by arresting the cell cycle. This was observed nearly two decades ago in colorectal cancer cell lines treated with the GUCY2C ligands, ST and uroguanylin, 8-Br-cGMP (a cell permeable cGMP), or the PDE inhibitor, zaprinast (86). Subsequently, inactivation of GUCY2C in mice was shown to accelerate epithelial cell cycle progression, specifically by releasing a block at the G1/S transition (81). These mice over-express epithelial cell cycle drivers (e.g., pRb, CDK4, cyclinD1, β-catenin) and under-express cell cycle suppressors (e.g., p27) (87). In turn, cGMP elevating agents, including ST, 8-Br-cGMP, and the PDE inhibitor, exisulind, increase transcription of the cyclin dependent kinase inhibitors, p21 and p27, which control this G1/S transition (85, 87, 88). PKGII-mediated phosphorylation and activation of the transcription factor, SP1, initiates transcription at the p21/p27 promoters (88).

**Figure 4:**
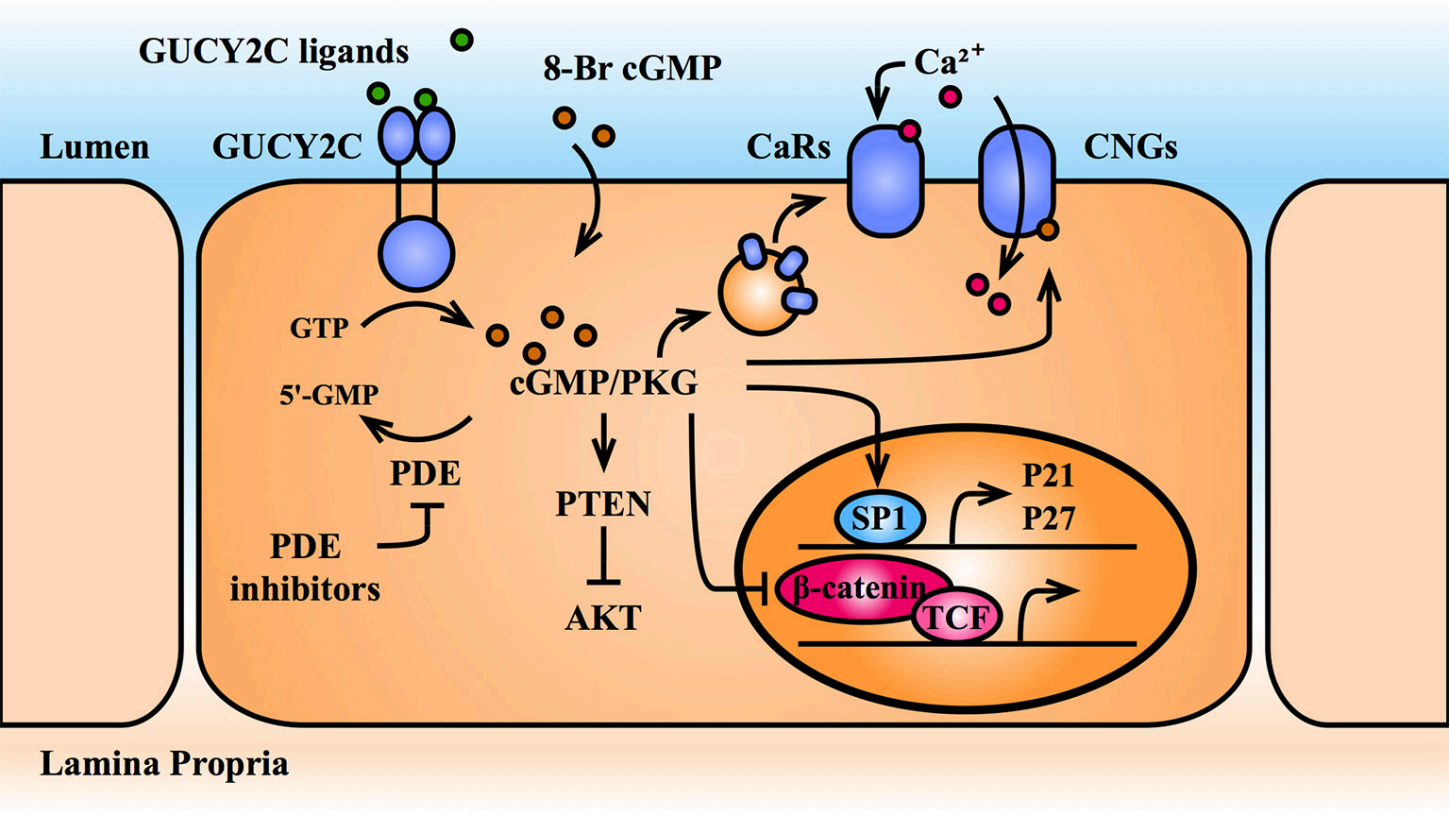
cGMP opposes cellular proliferation. cGMP elevating agents include GUCY2C agonists, PDE inhibitors, and cell-permeable 8-Br-cGMP. cGMP and its effector, PKG, oppose intestinal epithelial cell proliferation by upregulating nuclear transcription of cell cycle inhibitors (p21 and p27) and by opposing pro-proliferative transcription mediated by the β-catenin/TCF and Akt pathways. Further, cGMP recruits calcium-sensing G-protein coupled receptors (CaRs) to the plasma membrane and directly activates cGMP-gated calcium channels (CNGs), promoting Ca^2+^-mediated cytostasis. Akt, protein kinase B; CaR, calcium-sensing receptor; cGMP, cyclic guanosine monophosphate; CNG, cGMP-gated calcium channel; GTP, guanosine triphosphate; GUCY2C, guanylate cyclase C; p21, cyclin dependent kinase inhibitor 1A; p27, cyclin-dependent kinase inhibitor 1B; PDE, phosphodiesterase; PKG, protein kinase G; PTEN, phosphatase, and tensin homolog; SP1, specificity protein 1; TCF, T-cell factor; 5′-GMP, 5′-guanosine monophosphate; 8-Br-cGMP, 8-bromo-cGMP.

In addition to cell cycle arrest, cGMP signaling modulates other pathways involved in cell proliferation (Figure 4). For example, extracellular Ca^2+^ opposes proliferation through activation of plasma membrane-bound calcium-sensing receptors (CaRs), and entry through cyclic-nucleotide-gated ion channels. Stimulation of GUCY2C with ST recruits CaRs to the cell surface and activates cGMP-gated channels, resulting in Ca^2+^-mediated cytostasis (89). In addition, transcriptomic profiling of GUCY2C^+/+^ and GUCY2C^−/−^ mouse intestinal epithelia revealed activation of pro-proliferative circuits downstream of the serine/threonine kinase, AKT, including metabolic reprogramming to a neoplastic, glycolytic phenotype (87). Reconstitution of cGMP signaling mobilizes the phosphatase, PTEN, the canonical inhibitor of AKT, reversing this phenotype (87). Reports also suggest that cGMP directly opposes the proliferative transcriptional program of β-catenin/TCF (90). cGMP elevation through PDE5 and PDE10 inhibition reduced β-catenin accumulation, nuclear translocation, and downstream transcriptional activity by an unknown mechanism in multiple cancer cell lines (91, 92). It has been proposed that PKGII suppresses β-catenin/TCF transcription through activation of cJun N-terminal kinase (JNK) and the downstream forkhead box O transcription factor 4 (FOXO4) (93). In this model, activated-FOXO4 binds and recruits β-catenin to alternative DNA-binding sites, preventing its association with TCF and reducing TCF-mediated transcriptional output. However, it was later shown by the same group that PKGII suppresses JNK in mice (94). Furthermore, although there have been reports of TCF-independent recruitment of β-catenin to DNA, including by the FOXO family, a recent comprehensive analysis of β-catenin DNA-binding sites in mouse intestinal crypts revealed that TCF family members are universally required for β-catenin recruitment (95). Hence, the mechanisms by which cGMP signaling opposes proliferation remain debated and likely involve with several pathways.

### cGMP, genetic instability, and DNA damage repair

The intestinal epithelium is continuously exposed to DNA damaging agents. These include exogenous agents, such as radiation, microorganisms, and mutagenic substances in the lumen, as well as endogenous agents, such as reactive oxidative species (ROS) generated by metabolically-active crypt cells (96). Cells detect and respond to DNA damage by several mechanisms. The best-characterized guardian of genomic integrity, p53, activates a transcriptional program in response to DNA damage (97). Canonically, this begins with transcription of p21 to suspend the cell cycle and DNA replication, followed by activation of genes encoding DNA repair machinery, or if the damage is beyond repair, pro-apoptotic Bcl-2 proteins. Genetic instability, the corruption of normal DNA repair mechanisms and accumulation of mutations, is a hallmark of cancer and plays a central role in colorectal tumor progression (77, 98). Most sporadic colorectal tumors arise through a specific series of mutations, termed the adenoma-carcinoma sequence, beginning with APC, and followed by mutations in tumor suppressors such as p53 (60–70%) or oncogenes such as KRAS (40%) that enable transformation (77). Interestingly, only 10% of preneoplastic lesions progress to carcinoma over a 10 year period, as the accumulation of mutations required for tumorigenesis is slow (77). Genetic instability and disrupted damage sensing mechanisms accelerate the rate of mutation and provide a survival advantage to malignant cells.

Loss of APC predisposes for colorectal cancer partly because it suppresses oncogenic β-catenin/TCF-driven transcription, but also because APC regulates DNA repair and chromosomal stability (99, 100). APC shuttles between the cytoplasm, where it regulates the β-catenin destruction complex, and the nucleus, where it modulates the DNA base excision repair, double strand break repair, and replication fork dynamics (101–104). Furthermore, through interactions with EB1, a microtubule-binding protein, APC associates with the kinetochore in mitotic cells, regulating spindle assembly, orientation, and chromosome segregation (105). Cells harboring truncated APC mutants have defects in chromosome segregation (106) and APC^min/+^ mice exhibit tetraploidy (107). Indeed, a recent report highlighted the central importance of APC in intestinal tumor suppression using a doxycycline-inducible APC shRNA, enabling toggling of wild type APC. Elimination of APC in the context of p53 and KRAS mutations produced tumors, but removal of the shRNA, reconstituting APC in established tumors, rapidly reversed transformation and restored normal crypt-villus architecture (108).

cGMP signaling promotes DNA damage repair and opposes chromosomal instability in healthy tissue and in the context of APC defects. Elimination of GUCY2C from wild type and APC^min/+^ mice produces DNA double strand breaks (quantified by the marker phospho-γH2AX) and DNA oxidation (84, 109). This underlying environment of DNA damage in the absence of cGMP signaling predisposes to further accumulation of mutations. Indeed, tumors from APC^min/+^ mice lacking GUCY2C have a higher frequency of APC loss of heterozygosity than those with GUCY2C, reflecting genomic instability (84). Although the mechanism has yet to be fully defined, cGMP signaling contributes to genomic stability at least in part through metabolic reprogramming from glycolysis to oxidative phosphorylation (characteristic of proliferating vs. quiescent cells), decreasing ROS production and oxidative DNA damage in mice and cancer cell lines (87). Interestingly, deletion of CDX2, the intestinal transcription factor responsible for GUCY2C expression (110, 111), similarly potentiates tumor burden, chromosomal aberrations, and APC loss of heterozygosity (112). This reflects stimulation of mTOR, a downstream effector of AKT (112). Furthermore, it was recently reported that GUCY2C contributes to DNA integrity in part through a mechanism mediated by p53 (113). Elimination of GUCY2C from mice increased, while oral administration of the GUCY2C ligand, ST, reduced radiation-induced gastrointestinal toxicity in mice. cGMP signaling potentiated p53 activation in response to radiation injury, reducing DNA double strand breaks, abnormal mitotic orientation, and aneuploidy (characteristics of chromosomal instability). In summary, cGMP signaling potentiates DNA damage response mechanisms and promotes cellular quiescence, reducing susceptibility to chromosomal instability underlying tumor progression.

### cGMP, intestinal inflammation, and epithelial barrier integrity

The intestinal epithelium serves as both a selective conduit and a barrier between the luminal and systemic compartments. Transport between these compartments occurs by the transcellular route (via selective amino acid, electrolyte, and other nutrient transporters on the apical and basolateral surfaces of the enterocyte) or by the paracellular route, which is regulated by junctional complexes that bind the lateral walls of epithelial cells together (114). These junctional complexes are divided into three categories: desmosomes, adherens junctions, and tight junctions, the latter of which seals the paracellular space and is responsible for selective transport between cells. Numerous factors regulate junction complex integrity, particularly regulators of the inflammatory response (115–117). Endogenous anti-inflammatory cytokines, such as interleukin-10 (IL-10), promote barrier integrity (118). Others, such as tumor necrosis factor alpha (TNFα) and interferon gamma (IFNγ), key mediators of inflammation, increase barrier permeability through myosin light chain kinase (MLCK)-mediated phosphorylation of myosin light chain (MLC), leading to tight junction disassembly (119). Exogenous factors, such as alcohol and pathogenic microorganisms, also increase membrane permeability (114). Dysfunction of the epithelial barrier contributes to the pathology of numerous diseases, including inflammatory bowel disease (IBD), irritable bowel syndrome (IBS), sepsis, and autoimmune disease like celiac disease and type I diabetes (114, 117). Severe intestinal inflammation, such as IBD, also increases colorectal cancer risk (120).

Aside from the overt phenotype of intestinal secretory dysfunction, individuals harboring mutations in GUCY2C suffer from IBD (9, 10), suggesting that cGMP signaling regulates intestinal inflammation. Indeed, elimination of GUCY2C from mice produces an inflammatory phenotype associated with increased circulating and epithelial cytokines (109, 121, 122). In one study, intraperitoneal lipopolysaccharide injection (a bacterial endotoxin that provokes the immune response), produced greater proinflammatory gene expression (including TNFα and IFNγ) in colonocytes of GUCY2C^−/−^ relative to wild type littermates (122). Additionally, elimination of GUCY2C from the intestine of a genetic intestinal colitis model (IL10^−/−^) accelerated the onset of the disease (122). This suggests that homeostatic cGMP signaling reduces sensitivity to inflammatory stimuli. For example, mice exposed to dextran-sodium sulfate (DSS; a chemical model of intestinal inflammation mimicking IBD) and treated with plecanatide (a GUCY2C agonist) or sildenafil (a PDE5 inhibitor), were protected from inflammation compared to untreated mice (43, 123, 124). This effect was measured by epithelial histologic scoring, immune cell recruitment, expression of inflammatory cytokines, and inflammation-driven tumorigenesis (43, 123, 124).

cGMP signaling opposes intestinal inflammation at least in part through protection of epithelial barrier integrity. Elimination of GUCY2C in mice produces a phenotype of increased intestinal permeability, driven by MLC-mediated tight junction disassembly and increased basal levels of epithelial IFNγ, a canonical driver of intestinal permeability (121). Subsequent transcriptomic profiling of GUCY2C^−/−^ mouse epithelium also revealed decreased expression of 74 tight junction genes, including occludin, claudin-2, claudin-4, and JAM-A, contributing to loss of barrier integrity and susceptibility DSS-induced colitis (109). These changes were mediated by aberrant AKT signaling, which is opposed by cGMP signaling. A complementary mechanism was recently proposed, examining the role of reactive oxygen species in disrupting barrier integrity. Wang et al. suggest that cGMP signaling enhances barrier integrity through activation of the transcription factor, FOXO3a and its downstream antioxidant transcriptional targets (125). FOXO3a is phosphorylated and inactivated by AKT. Treatment of colon cancer cells, human biopsy specimens, and mice with 8Br-cGMP or the PDE5 inhibitor, vardenafil, suppressed AKT signaling, activated FOXO3a-mediated transcription of antioxidant species, and enhanced barrier integrity in the DSS-colitis model. These effects were abolished in PKGII^−/−^ animals, confirming the role of cGMP. Collectively, several laboratories have confirmed that cGMP signaling promotes intestinal barrier integrity and opposes intestinal inflammation. The extent to which these effects are mediated changes in cytokine expression, regulators of tight junction assembly, expression of junction components, or potentiation of antioxidant species remains an open-ended question.

### cGMP and the intestinal microbiome

Beyond its role in regulating fluid transport and nutrient absorption, the human intestine serves as a host for the densest population of microorganisms in the body, over 10^11^ microbes/mL by intestinal volume (126). The gut microbiome consists of over 1,000 species, varying in proportion from individual to individual depending on age, diet, geographic location, genetics, and other factors, which we have only begun to dissect since in the advent of large scale sequencing techniques (127, 128). Commensal bacteria, predominantly of the *bacteroidetes* and *firmicutes* phyla, thrive in the nutrient-rich environment provided by the intestinal epithelium. In turn, they complement gaps in host metabolic pathways, such as the fermentation of indigestible carbohydrates and synthesis of short chain fatty acids, a key energy source and signaling molecule for the epithelium (126, 128, 129). Beyond metabolic commensalism, gut bacteria defend against colonization by pathogenic species. These bacterial defense mechanisms occur indirectly through stimulation of the host immune response, and directly through nutrient competition and release of bactericidal small molecules (126, 130). For example, bacterial synthesis of short chain fatty acids opposes infection by enteropathogenic *E. coli* and virulence gene expression by *S. Typhimurium* in the colon (131, 132).

Alterations in diversity and composition of the intestinal flora, termed dysbiosis, characterize several intestinal diseases, including IBD and colorectal cancer. Whether these changes are a cause or consequence of disease remains an active area of research. However, mice treated with antibiotics, or housed in germ-free environments, exhibit intestinal mucus thinning, susceptibility to colitis, and acceleration of tumorigenesis, indicating that bacterial factors play a driving role (133–136). Chronic inflammation (e.g., IBD) is a risk factor for colorectal cancer, and bacterial species may contribute to tumorigenesis by producing an inflammatory state. Enrichment of specific bacterial species in the intestines of colorectal cancer patients, such as pro-inflammatory *Fusobacterium* and *Enterococcaceae*, and loss of anti-inflammatory butyrate-producing strains, such *Roseburia* and *F. prausnitzii*, alter the epithelial microenvironment and increase tumor susceptibility (137). Further, pro-carcinogenic species, including strains of *E. faecalis*, and *E. coli*, produce ROS and genotoxic virulence factors that drive mutations underlying transformation (137). Indeed, it was recently reported that patients with the hereditary colon cancer syndrome, FAP, harbor patches of *E. coli-*, and *B. Fragilis-*enriched biofilms, which are absent in normal individuals (138). These species secrete the toxins colibactin and B. fragilis toxin, respectively, which increase levels of inflammatory cytokines, DNA damage, and tumor onset in mice (138).

Epithelial cGMP has recently emerged as a regulator of microbiome composition, particularly through modulation of epithelial mucus properties. The colonic mucus is comprised two layers – (1) a sterile inner layer, rich in secreted immunoglobulin A and bioactive molecules (e.g., trefoil factor peptides, restin-like molecule b), that protects the epithelium from direct bacterial contact, and (2) an outer layer home to bacterial flora (126, 139). The mucus matrix is organized around the glycoprotein, mucin 2, secreted by epithelial goblet cells, which provides attachment sites and nutrition to commensal bacteria in the outer layer (139). It has been hypothesized that cGMP-mediated regulation of mucus hydration and pH through apical CFTR and NHE3 channels regulates bacterial colonization of the epithelial surface (140, 141). Indeed, elimination of GUCY2C from mice alters the composition of bacterial flora detected in the stool (140). Further, compromised barrier integrity in these mice increased susceptibility to systemic dissemination of the murine enteric pathogen, *C. rodentium* (140). Mice lacking GUCY2C also were more susceptible to a bacterial species that actively invades enterocytes, *S. enterica*, due to thinning of the protective mucus layer (141). In turn, administration of a GUCY2C agonist reduced bacterial adhesion and invasion. These findings support the notion that cGMP-mediated modulation of mucus hydration regulates bacterial colonization, and in turn, the relative proportions of commensal vs. pathogenic species.

cGMP signaling, microbiome composition, and colorectal cancer intersect in the long-recognized inverse relationship between colonization with diarrheagenic *E. coli* and incidence of colorectal cancer. Geographic regions with endemic enterotoxgenic *E. coli* (ETEC, responsible for Traveler's diarrhea), which produce the virulence factor and GUYC2C agonist, ST, have far lower rates of colon cancer (142). ST stimulation of GUCY2C arrests cell proliferation (86, 89, 142), suggesting an intriguing hypothesis that chronic ETEC colonization confers tumor resistance. Our group recently confirmed a role for chronic ST-exposure in tumor prevention. Mice colonized for 18 weeks with ST-producing *E. coli*, mimicking chronic ST exposure in endemic regions of the world, developed a 50% lower tumor burden in response to the carcinogen, azoxymethane, than mice colonized with ST-negative *E. coli* (143). This finding reinforces the role of the GUCY2C-cGMP signaling axis, as well as the role of microbiome composition, in tumor susceptibility.

### cGMP and epithelial-mesenchymal cross talk

Intestinal development and homeostasis rely on reciprocal signaling between the epithelium and underlying lamina propria. Derived from embryonic mesoderm, the lamina propria consists of acellular (extracellular matrix) and cellular [fibroblasts, pericytes, stromal stem cells, smooth muscle cells; (144)] elements that provide structural support and paracrine cues to the epithelium. Mesenchymal cells regulate epithelial proliferation and senescence, maintain and restrict the stem cell niche, and remodel the extracellular matrix (145). Under normal conditions, stromal fibroblasts remain in a quiescent state, secreting extracellular matrix proteins, matrix-modulating enzymes, and soluble growth and differentiation factors that maintain the underlying stroma architecture and promote epithelial differentiation. For example, fibroblasts surrounding the crypt base secrete Wnt molecules (Wnt2b, 4, 5a, 5b) and BMP antagonists (gremlin-1, gremlin-2, chordin-like 1) that have receptors on the epithelium and drive proliferation in the crypt (65, 146, 147, 148). They also restrict the stem cell niche in a vertical gradient through secretion of BMPs and Wnt antagonists to prevent β-catenin signaling outside of the normal proliferating zone (65, 72, 146, 147, 149). Fibroblasts also respond to epithelial injury (including mechanical stress, reactive oxidative species, inflammatory cytokines, or growth factors), converting to metabolically active myofibroblasts, which engage in matrix remodeling necessary for wound repair (144, 147). The primary stimulus driving the conversion of fibroblasts to myofibroblasts is transforming growth factor beta (TGFβ), secreted by the overlying epithelium. Resolution of injury repair and decline of TGFβ secretion results in myofibroblast apoptosis and/or reversion to a quiescent phenotype.

Pathological conditions, including chronic inflammation and neoplastic transformation, promote the recruitment of activated fibroblasts in a mutually reinforcing feedback loop. Secretion of TGFβ by injured, inflamed, or neoplastic epithelium activates fibroblasts, inducing changes in their proliferation, migration, adhesion, secretory, and matrix remodeling properties (145, 150–152). In turn, activated fibroblasts produce a stromal environment rich in extracellular matrix and secreted growth factors that are conducive to tumor growth, termed desmoplasia. Desmoplastic stroma has unique properties that promotes tumor invasion and metastasis, including remodeling of the normal Wnt and BMP gradients that define crypt architecture (145, 153, 154). Cancer-associated fibroblasts also directly promote tumorigenesis through the secretion of inflammatory cytokines and growth factors, such as hepatocyte growth factor (HGF), which is recognized by epithelial MET proto-oncogene receptor tyrosine kinase (c-MET) and promotes proliferation and invasion (145, 153).

cGMP signaling opposes epithelial-mesenchymal interactions underlying tumorigenesis. Several studies have described mechanisms by which intestinal cGMP signaling inhibits cancer cell migration, invasion, and microenvironment remodeling (155–159). cGMP suppresses the release of matrix metalloproteinases (MMPs; enzymes that cleave extracellular matrix components) by colon cancer cells, and was shown to prevent metastatic seeding of these cancer cells in mice (156). Further, loss of cGMP signaling in colon cancer cells promotes the assembly of actin-based motility organelles (filopodea) and invasion organelles (invadopodia) involved in tumor cell migration (157). PKG-mediated phosphorylation of vasodilator-stimulated protein (VASP), an actin-binding protein, opposes this cytoskeletal remodeling (157). Finally, silencing GUCY2C in mice and human cancer cells drives AKT-dependent secretion of TGFβ by the epithelium, producing fibroblast activation and a desmoplastic phenotype characteristic of early transformation (158). In turn, activated fibroblasts secrete HGF, reciprocally driving epithelial proliferation. Collectively, cGMP signaling opposes matrix remodeling and a cellular-invasion phenotype.

Beyond its role as an intracellular second messenger, cGMP also acts as a paracrine signaling molecule in the intestine. Activation of GUCY2C produces intracellular cGMP accumulation, as well as cGMP release into the extracellular environment (160–162). This extrusion is mediated by the membrane anion channel, multi-drug resistance protein 4 (MRP4), expressed on the apical and basolateral membranes of the epithelium (162, 163). Extracellular cGMP promotes analgesia by acting on visceral nociceptive neurons, and as such, the GUCY2C signaling axis has been targeted for the treatment of pain in constipation-predominant irritable bowel syndrome (IBS-C) (160, 161, 164). Other roles for cGMP in the intestinal stroma are unknown. It is tempting to speculate that given the various tumor-suppressive roles of cGMP signaling, pathological conditions that diminish extracellular cGMP could create a local microenvironment susceptible to transformation. However, its role as a paracrine tumor suppressor remains purely hypothetical because a cGMP receptor or cGMP uptake transporter have yet to be identified, and its extracellular mechanisms of action remain elusive.

## cGMP dysregulation in colorectal cancer and therapeutic implications

Colorectal cancer remains the second leading cause of cancer death and fourth most incident cancer in the United States (165). Genetic alterations underlying tumorigenesis have been well defined; namely, the driving mutations in APC and β-catenin, which lift a block on proliferation along the crypt-villus axis (77). Furthermore, certain risk factors such as chronic inflammation (i.e., IBD), smoking, and obesity predispose patients to the development of tumors. Yet, the underlying changes in the intestinal epithelial microenvironment that tip the homeostatic balance in favor of tumorigenesis remain poorly understood. Genetic mutations represent an irreversible phenomenon, and the standard of care remains surgical and chemotherapeutic approaches to eliminate transformed tissue. Hence, the identification of reversible factors contributing to the earliest stages of transformation are needed.

### Cell-autonomous suppression of cGMP signaling in colorectal cancer

**Figure 5:**
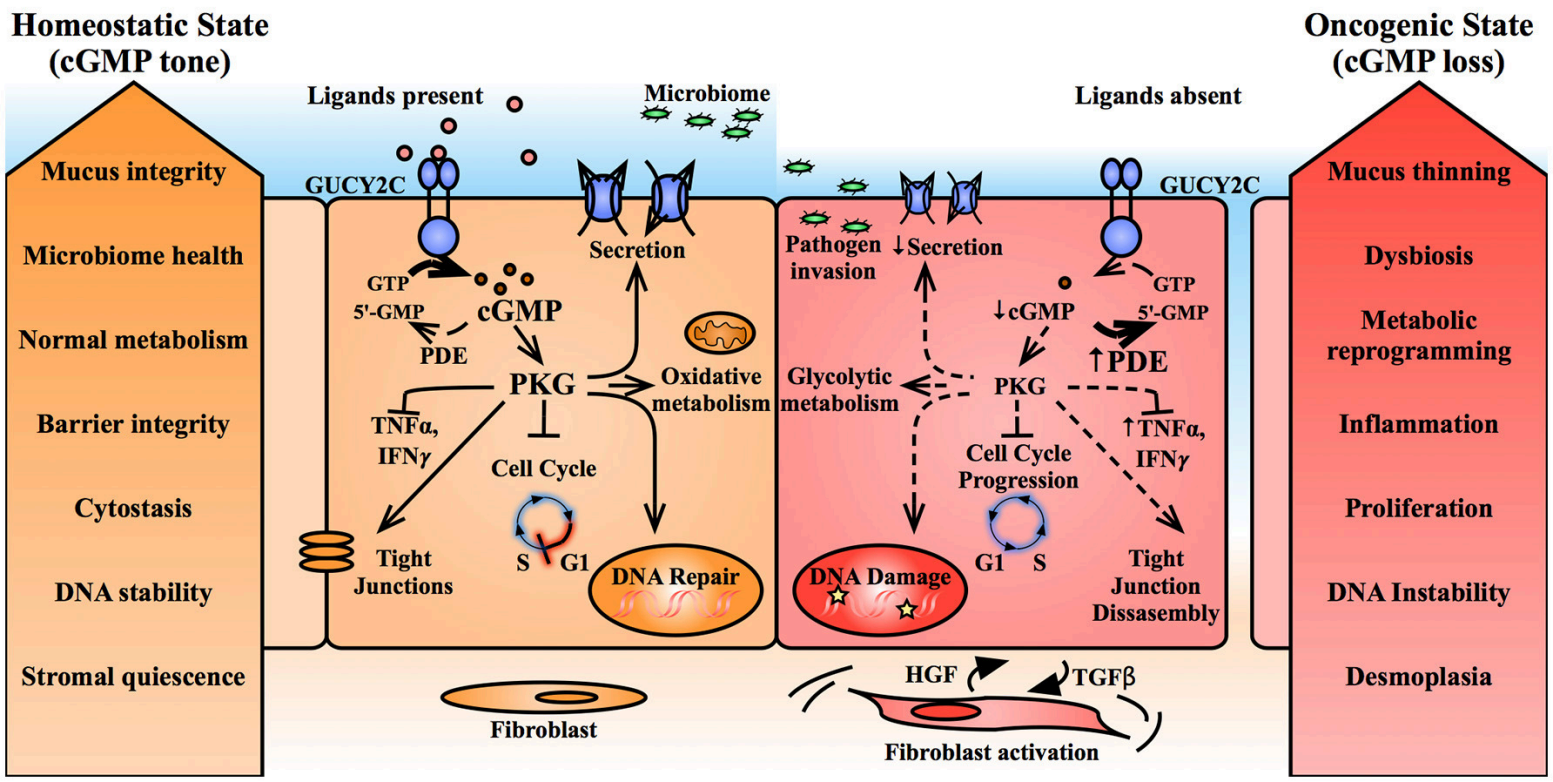
Suppression of cGMP signaling promotes epithelial transformation. Basal cGMP tone in the intestinal epithelium promotes epithelial homeostasis. cGMP, through its effector, PKG, regulates several downstream mechanisms including mucus hydration, metabolism, inflammation, barrier integrity, DNA damage sensing, and cell cycle progression. In contrast, intestinal transformation is characterized by upregulation of phosphodiesterases and suppression of GUCY2C paracrine ligands, reducing intracellular cGMP and producing an epithelial phenotype predisposed to cancer progression. cGMP, cyclic guanosine monophosphate; GTP, guanosine triphosphate; GUCY2C, guanylate cyclase C; HGF, hepatocyte growth factor; IFNγ interferon gamma; PDE, phosphodiesterase; PKG, protein kinase G; TGFβ, transforming growth factor beta; TNFα tumor necrosis factor alpha; 5′-AMP, 5′-adenosine monophosphate; 5′-GMP, 5′-guanosine monophosphate.

cGMP has emerged as a key regulator of intestinal circuits that oppose tumorigenesis (Figure 5). As such, suppression of cGMP signaling is a common thread in colorectal cancers and may be necessary for tumorigenesis. Indeed, a recent analysis of mRNA and long non-coding RNA expression in tumor vs. normal tissue samples on the TCGA database identified the PKG-cGMP pathway among the top regulated gene networks (166). One mechanism of cGMP suppression is altered intracellular expression of cGMP axis elements, resulting in loss of cGMP signaling in cancer cells. For example, upregulation of PDEs accelerates hydrolysis of cyclic nucleotides. PDE5 elevation has been observed in human colon cancer cell lines and tumor samples compared to normal tissue, and PDE10 elevation has been observed in human cell lines, biopsy specimens, and tumors from APC^min/+^ mice (58, 90, 124, 167). Another study found that expression of PDE4B (which preferentially degrades cAMP) was elevated in histologically normal-appearing intestinal epithelium from colorectal cancer patients, suggesting that cyclic nucleotide dysregulation occurs early in transformation, preceding other histologic markers (57). Suppression of the cGMP effector, PKGI, has also been observed in colon tumor specimens compared to normal tissue, contributing to angiogenesis in tumor xenografts (168, 169).

Inhibition, rather than changes in expression of cGMP signaling elements also contributes to silencing of the signaling axis. C-src, a tyrosine kinase overexpressed in colorectal cancer, phosphorylates tyrosine 820 on the catalytic domain of GUCY2C, inhibiting receptor activation (170). An alternative mechanism of receptor silencing may involve removal from the cell surface and sequestration in subcellular compartments, which was recently observed by immunohistological staining of multiple gastrointestinal malignancies (171). Whether changes in localization regulate cGMP generation remains unknown. Hence, through various mechanisms, silencing the tumor-suppressive properties of cGMP signaling appears to be a common feature of colorectal cancer.

### GUCY2C paracrine hormone loss in colorectal cancer

The aforementioned examples focus on cell-autonomous mechanisms of modulating of intracellular cGMP signaling in tumorigenesis. Another intriguing paradigm recognizes the role of cGMP signaling in intercellular communication via the secretion of GUCY2C ligands that act in an autocrine and paracrine fashion. The GUCY2C ligands, guanylin, and uroguanylin, are among the most commonly lost gene products in colorectal cancers, and this loss is conserved between mice and humans (172–176). For example, in a study of 300 patient tumor samples, >85% exhibited loss of guanylin (the colonic hormone) mRNA and protein expression compared to matched normal adjacent tissue (176). Ligand loss is also observed in the context of diet-induced obesity and intestinal inflammation, conditions which predispose to the development of colorectal cancer, and may represent a mechanistic link between these risk factors and tumorigenesis (122, 177, 178). Elimination of guanylin from mice results in loss of epithelial cGMP, producing crypt hyperplasia (80), and ligand reconstitution through oral administration or by transgenic expression opposes tumorigenesis (174, 177). Importantly, the receptor, GUCY2C, is retained in transformed tissue, despite the loss of its ligands (16, 171, 174, 179). Collectively, these findings underlie the *paracrine hormone hypothesis* of colorectal cancer (180), which suggests that guanylin insufficiency silences the tumor suppressive properties of the GUCY2C-cGMP axis, producing a microenvironment conducive to transformation.

### Colorectal cancer prevention by restoring the cGMP axis

Activation of cGMP signaling, thereby promoting epithelial homeostasis and restoring its tumor suppressive function represents an enticing approach to cancer prevention, potentially overcoming irreversible genetic mutations in APC or β-catenin. The enzymes responsible for cGMP generation and degradation can be targeted for pharmacological regulation, for example with GUCY2C agonists or PDE inhibitors. Among the earliest demonstrations of the efficacy of targeting the GUCY2C-cGMP axis for tumor prevention, Shailubhai et. al. observed a reduction of tumor burden in APC^min/+^ mice fed uroguanylin in the diet (174). Since then, cGMP-elevating agents, including PDE inhibitors (Table 1) and GUCY2C agonists (Table 2) have been shown to oppose cellular proliferation, genomic instability, barrier dysfunction, inflammation, dysbiosis, desmoplasia, and other factors discussed above that contribute to tumorigenesis.

**Table 1:** PDE inhibitors shown to oppose tumorigenic cell circuits.

	**Specificity**	**Functions Regulated**	**Models**
Sulindac sulfone (Exisulind)	PDE5	Apoptosis, (181) cell proliferation, (88) polyp multiplicity (182–184)	cells, mice, humans
Sulindac benzylamine	PDE5	Cell proliferation and apoptosis (167)	cells
Sulindac sulfide	PDE5	Cell proliferation and apoptosis (185)	cells
Sildenafil	PDE5	Polyp multiplicity, (186) colitis, (124) inflammation-induced polyps (187, 124)	mice
Vardenafil	PDE5	Cell proliferation (94), colitis (94), redox stress (125)	cells, mice
Zaprinast	PDE5, 6, 9, 11	Cell proliferation (86)	cells
ADT-094	PDE5, 10	Cell proliferation (91)	cells
Papaverine	PDE10	Cell proliferation (91, 58)	cells
PQ-10	PDE10	Cell proliferation (58)	cells
Pf-2545920	PDE10	Cell proliferation (58, 92)	cells

**Table 2:** GUCY2C agonists shown to oppose tumorigenic cell circuits.

	**Structural Analog**	**Functions Regulated**	**Models**
ST		Ca^2+^ conductance, (142, 89) cell proliferation, (85, 89) matrix remodeling and invasion, (156, 157) DNA damage sensing, (113) fibroblast activation, (158) tumor metabolism, (87) colitis and barrier permeability, (109) pathogen defense, (141) carcinogen-induced tumorigenesis, (143)	cells, mice
Guanylin		matrix remodeling and invasion, (156) colitis and barrier permeability, (109) obesity-induced tumorigenesis (177)	mice
Uroguanylin		Cell proliferation, (86) matrix remodeling and invasion, (156) polyp multiplicity (174)	mice
Linaclotide	ST	Polyp multiplicity, (186) cGMP efflux, (162) intestinal pain (160)	cells, mice, humans
Plecanatide	Uroguanylin	Colitis, (43) inflammation-induced dysplasia (123)	mice
Dolcanatide	Uroguanylin	Colitis (43)	mice

Supporting the feasibility of targeting the cGMP axis for tumor prevention, several cGMP elevating agents have been shown to oppose colorectal tumorigenesis in clinical and pre-clinical models. Early trials tested the PDE5 inhibitor, exisulind, in patients with FAP and sporadic colorectal adenomas (182–184). Exisulind, the sulfonated derivative of the NSAID, sulindac, produces intracellular cGMP accumulation, driving caspase-mediated apoptosis in cancer cells (181, 188). In patients, exisulind treatment produced tumor cell apoptosis and polyp regression, but significant hepatic toxicity at therapeutic doses proved insurmountable (182–184). Further, the antineoplastic mechanism of action has been questioned (189), leading to interest in alternate cGMP elevating agents with more desirable safety profiles. Recent studies have turned to agents already FDA-approved for other disorders. These include the PDE5 inhibitor, sildenafil, approved for the treatment of erectile dysfunction and pulmonary hypertension (53), and the synthetic GUCY2C ligands, plecanatide and linaclotide, which target the secretory function of GUCY2C to treat chronic idiopathic constipation and constipation-predominant irritable bowel syndrome (28). Recent reports showed that sildenafil and linaclotide administered orally in water reduced tumor multiplicity in the APC^min/+^ mouse (186). Furthermore, in mouse models of carcinogen-driven (azoxymethane) and inflammation-driven (DSS) tumorigenesis, sildenafil and plecanatide reduced the incidence of polyps and dysplastic lesions (123, 124, 187). These promising preclinical reports support approaching tumor prevention through reconstitution of the silenced GUCY2C-cGMP signaling axis.

While clinical translation of cGMP-elevating agents for tumor prevention is a logical next step, several questions remain to be answered regarding the mechanism of tumor suppression by the GUCY2C-cGMP axis. One area of debate is the nature of colorectal cancer inception, and where along the transformation continuum cGMP exerts its effects. It remains unclear if cGMP elevating agents oppose the initial drivers of tumorigenesis, for example by opposing genetic instability and therefore avoiding the sequential accumulation of mutations beginning with APC loss. Alternatively, healthy cGMP tone may promote a homeostatic microenvironment that suppresses proliferative signaling, inflammation, and desmoplasia, thereby preventing cancer progression in spite of APC/β-catenin mutations. Another open debate is the nature of the GUCY2C-cGMP axis suppression in cancer, and its implications for therapeutic reconstitution of cGMP signaling. PDEs are overexpressed in transformed tissue, albeit by an unknown mechanism, suggesting that cGMP loss is a cell-autonomous result of transforming mutations. In turn, PDE inhibitors would effectively elevate epithelial cGMP and oppose tumor progression. An alternative view recognizes that endogenous GUCY2C ligands are suppressed early in transformation (again, by a mechanism yet to be defined), suggesting that a paracrine field of GUCY2C silencing is responsible for cGMP loss and tumor susceptibility. This latter paradigm supports the reconstitution of cGMP signaling with exogenous ligand replacement, and forms the basis for clinical trials exploring oral GUCY2C agonists as a chemopreventative strategy in humans (190). The relationship between the GUCY2C-cGMP axis and colorectal cancer inception will undoubtedly become clearer in the coming years, and these molecular insights will ultimately provide a mechanistic framework for tumor prevention.

## Conclusion

The GUCY2C-cGMP signaling axis has emerged as a key regulator of epithelial homeostasis in the intestine. Initially described as a the regulator of fluid and electrolyte secretion, cGMP is now recognized for its roles in modulating epithelial proliferation, DNA integrity, barrier function, microbiome composition, epithelial-mesenchymal cross talk, and other aspects of epithelial function. Dysregulation of these circuits underlies intestinal transformation, and perhaps unsurprisingly, loss cGMP signaling has emerged as a common feature of colorectal tumors. The precise role of cGMP signaling in the pathophysiology of colorectal cancer remains an open-ended question, but its tumor-suppressive properties are diverse. As such, suppression of cGMP signaling may be a necessary step in tumorigenesis because it lifts a block on proliferation, microenvironment remodeling, and the accrual of DNA mutations necessary for transformation. Supporting this notion, endogenous GUCY2C activating ligands are lost in early in transformation, and also from chronically inflamed epithelium, suggesting a mechanistic basis for this recognized risk factor for colorectal cancer. Preclinical data from several laboratories demonstrates that re-activation of cGMP signaling opposes tumor formation, and the availability of FDA-approved cGMP-elevating agents underscores the tractability of this approach. Given these observations, the GUCY2C-cGMP axis represents a logical, mechanism-based target for colorectal cancer prevention.

## Author contributions

JR wrote the manuscript with input, critical feedback, and revisions by SW.

### Conflict of interest statement

SW is the Chair (uncompensated) of the Scientific Advisory Board and a member of the Board of Directors of Targeted Diagnostics &amp; Therapeutics, Inc., which has a license to commercialize inventions arising from his work. Also, he receives research funding from, and has been a compensated speaker for, Synergy Pharmaceuticals, Inc. Further, he is Chair of the Board of Directors of Feelux Company, Ltd. The authors have no other relevant affiliations or financial involvement with any organization or entity with a financial interest in or financial conflict with the subject matter or materials discussed in the manuscript apart from those disclosed.
